# The effect of conservative non-pharmacological interventions on the management of urinary incontinence in older adults living with frailty: Systematic review and meta-analysis

**DOI:** 10.1371/journal.pone.0322742

**Published:** 2025-05-14

**Authors:** Maureen O’ Callaghan, Katie Robinson, Aoife Whiston, Morgan Senter, Amanda M. Clifford

**Affiliations:** 1 School of Allied Health, Faculty of Education and Health Sciences, University of Limerick, Limerick, Ireland; 2 Ageing Research Centre, Health Research Institute, University of Limerick, Limerick, Ireland; 3 Irish World Academy of Music and Dance, University of Limerick, Limerick, Ireland; University Hospital Cologne: Uniklinik Koln, GERMANY

## Abstract

**Background:**

Urinary incontinence (UI) is highly prevalent among older adults with frailty, impacting function, quality of life and risk of long-term care admission.

**Objective:**

To systematically review and synthesise the results of randomised controlled trials (RCTs) investigating the effect of conservative non-pharmacological interventions on the management of UI in older adults aged ≥ 65 years living with frailty.

**Methods:**

Five databases (Cochrane Library, Medline (EBSCO), CINAHL (EBSCO), Embase (OVID), PsycINFO (EBSCO)) were searched from inception to April 2024 for RCTs that evaluated conservative non-pharmacological interventions for UI in older adults living with frailty. Two independent reviewers screened records, assessed methodological quality using the Cochrane Risk of Bias (RoB 2.0) Tool and Level of Evidence was summarised using GRADE guidelines. A meta-analysis using a random-effects model or narrative synthesis were performed as appropriate.

**Results:**

Twelve RCTs, including 1,580 participants, with medium to high risk of bias were included. Conservative non-pharmacological interventions (categorised as single component or multicomponent interventions) resulted in a non-statistically significant reduction of objective measures of UI (6 RCTs, *g* = -0.39, *p* = 0.090; pooled effect size, with CI = -0.39 [-0.832, 0.060], *I*^*2* ^= 85.26%, with very low certainty of evidence). Improvements in functional ability were not found to be statistically significant (5 RCTs, *g* = 0.20, *p* = 0.39, pooled effect size, with CI = 0.20 [- 0.251, 0.642], *I*^*2* ^= 85.87%, and very low certainty of evidence). The interventions did not result in adverse events. Studies did not evaluate caregiver quality of life.

**Conclusions:**

Very low-quality evidence found that conservative non-pharmacological interventions had beneficial but not statistically significant effects on objective UI and functional ability. Due to the high incidence of intervening illnesses and mortality in older adults living with frailty, it is recommended that future studies assess the effect of implementing tailored interventions addressing modifiable risk factors using more appropriate study design and outcome measures.

**Registration:**

This review was prospectively registered on the International Prospective Register of Systematic Reviews, PROSPERO (CRD42022316287; https://www.crd.york.ac.uk/prospero/).

## Introduction

Urinary incontinence (UI) is defined by the International Continence Society as ‘any involuntary leakage of urine’ [1]. The prevalence of UI is up to three times higher in women than men and increases with age [2]. More than 40% of women aged ≥ 70 years report UI, which was found to increase up to 70% in nursing home residents and older adults in hospital [2]. Ageing can lead to physiological changes in the lower urinary tract (LUT) and brain including the development of white matter hyperintensities, which can contribute to the increase in UI with ageing [3,4]. However, UI should not be thought of as an inevitable part of ageing [5]. UI can be a taboo subject and is thus underreported; as identified in an Irish study (TILDA), which found a large proportion (40%) of patients with UI did not disclose it to a health care professional [6].

Older people with frailty are the fastest growing group affected by incontinence [1]. Frail older persons are described as those over the age of 65 with a clinical presentation defined by the Mitniski and Rockwood accumulated deficits model or by Frieds’ phenotypic model [1]. Incident UI is a known marker of frailty, with UI being twice as prevalent in older persons with frailty versus non-frail older persons [7,8].

UI is considered a geriatric syndrome, with multiple risk factors and comorbidities across multiple organ systems external to the LUT, the ageing LUT itself, and medications combining to inhibit the individuals’ ability to remain continent [3,4,9]. In a large sample of Irish adults aged ≥50 years, multimorbidity at baseline was associated with a twofold increase in risk for urinary incontinence onset at 2-year follow-up [10]. The consequences of UI in older persons with frailty include reduced function, falls and fractures, social isolation, low mood, caregiver burden and long-term care [1,11–13]. UI in nursing home residents’ results in a greater risk for mortality than in residents without UI [14].

Conservative non-pharmacological interventions for UI are recommended as the first line of interventions by the European Association of Urology (EAU) guidelines [15], and the NICE guidelines on UI in women [16], as the first line of treatment for all populations [17] and by the 7^th^ International Consultation on Incontinence (ICI) guidelines [1] for older adults with frailty. Treatment of UI in older adults living with frailty aims to address modifiable risk factors using individually tailored integrated multidisciplinary interventions collaboratively agreed with the patient and caregiver [1,18]. Current recommendations advise caution or avoidance of pharmacological management, due to a lack of evidence demonstrating efficacy, tolerability, and safety [1,18]. There is a high risk of adverse events, particularly with anticholinergic drugs, which can impair cognitive function [1,4,18,19]. However, some evidence exists for the use of antimuscarinic medications in the management of overactive bladder (OAB), associated with dry or wet urgency incontinence in medically complex older adults [1,18]. There is also a high risk of post-op complications, mortality, and requirement for repeat repairs from surgical management of UI in older adults with frailty [1,18,20].

Consequently, there is a need for conservative non-pharmacological interventions for UI in older adults living with frailty. Existing conservative interventions for UI include educational interventions, lifestyle and behavioural advice, psychological therapies, physical therapies, mechanical devices, and complementary therapies as described in a recent Cochrane overview of Cochrane systematic reviews for conservative management of UI in women aged ≥ 18 years old [21]. There is evidence for the effectiveness of some of these interventions in older women (aged > 65 years), as established in a systematic review of systematic reviews by Kilpatrick and colleagues which included 33 systematic reviews encompassing 27 primary trials [19]. In this review, group exercise therapy for stress incontinence, along with behavioural interventions that include bladder training, pelvic floor muscle training, and practical strategies for managing stress and urgency incontinence, were found to be beneficial in addressing urinary incontinence in older women [19]. However, the applicability of these findings to frail older adults is uncertain, as some trials for UI interventions exclude this population due to concerns about their ability to participate in or adhere to interventions [22]. Furthermore, older adults with frailty may have many co-morbidities that can contribute to incontinence, and as these accumulate, severity of frailty increases, and this may impair response to treatment [4] in contrast to non-frail populations [4]. Thus, there are unanswered questions regarding the most effective UI treatment strategies for older adults, both men and women aged 65 years and over with frailty [1,18,23]. Addressing this gap is crucial for informing frailty-guided clinical care, which is increasingly recommended for older individuals with frailty [24]. Furthermore, a review focusing solely on randomised controlled trials of UI interventions for older adults with frailty is warranted as the potential for bias is likely to be greater for non-randomised studies compared to randomised trials when evaluating the effects of interventions [25]. Of the 27 trials in the review by Kilpatrick and colleagues [19], four were non-randomised quasi-experimental study designs, five were pre/post intervention study designs, one was a prospective case series, and the remainder were randomised controlled trials.

Given the prevalence of urinary incontinence in older adults aged 65 years and over with frailty and the impact of urinary incontinence on function, quality of life and rates of admission to residential care, a comprehensive, contemporary, and robust systematic review to evaluate the totality of the evidence in relation to the effectiveness of non- pharmacological conservative interventions for urinary incontinence in older adults with frailty is warranted.

## Materials and methods

### Design

A systematic review and meta-analysis were conducted to address the research question. This systematic review was prospectively registered on the International Prospective Register of Systematic Reviews, PROSPERO (CRD42022316287) (S1 Appendix) in April 2022. The PRISMA guidelines were followed in the conduct and reporting of this systematic review (S1 Checklist, S2 Checklist) [26].

### Search strategy

Five electronic databases were searched from inception by the lead author in April - June 2022 and repeat searches were conducted in May 2023 and April 2024 (The Cochrane Library, Medline (EBSCO), CINAHL (EBSCO), Embase (OVID), PsycINFO (EBSCO)). The search strategy S1. File was developed with an experienced librarian based on the Cochrane Incontinence Group list for search strategies used for the Cochrane Incontinence Specialised register and included core terms of frail older adults, urinary incontinence, and randomised controlled trials. In addition, reference lists of included RCTs were reviewed to identify other potentially relevant RCT’s for inclusion.

Studies meeting the following criteria were included: Peer reviewed articles published in the English language, with no time limits on publication. **Population**: adults 65 years and older with frailty who experience bothersome urinary incontinence of non-neurogenic and non-cancer cause. In studies with mixed populations, the whole study was included if 80% of the study population could be classified as frail. Participants from the community, acute hospital or residential care settings were included. Frailty was determined using the International Consultation on Incontinence (ICI) definition of frail older persons as those over the age of 65 with a clinical presentation defined by the Mitnitski and Rockwood accumulated deficits model or by Frieds’ phenotypic model [1]. Cognitive and physical impairments, presence of multimorbidity, and residence in nursing homes or residential care were also included as defined in our inclusion criteria. **Intervention:** Conservative non-pharmacological interventions implemented as single modalities or in multiple combinations as part of a multicomponent approach delivered by any healthcare professional were included. Conservative non pharmacological interventions were categorised as per the Cochrane Incontinence Group into lifestyle (dietary and fluid modifications, constipation management, smoking cessation, and weight loss) educational (education to patients, staff and family carers), behavioural and psychological (behaviour training, voiding programmes, bladder training, bladder diaries, alarms), and physical interventions (physiotherapy including pelvic floor muscle training/exercises, biofeedback, electrical stimulation, vaginal cones, exercise therapy, and occupational therapy including functional training, and environmental modifications) and outlined in our PROSPERO Protocol (S1 Appendix). The conservative management strategies for UI included in our review included prompted voiding as a single modality intervention and multicomponent interventions (including a range of lifestyle, educational, behavioural and psychological and physical interventions).

**Comparison** Any comparator intervention of no treatment, usual care, placebo, or sham treatments were considered. Studies comparing two or more interventions to each other, or two or more interventions grouped together versus usual care were also included. **Outcomes:** The primary outcome was a change in episodes of urine leakage assessed using objective measures of reduced wet episodes. Subjective measures of change of urine leakage, patient quality of life and informal caregivers’ quality of life were also measured. Additional outcomes were incidence of adverse effects, functional ability and self-reported improvement. **Study design:** All randomised controlled trials (RCTs) including cluster RCTs or quasi-RCTs, were included.

Studies were excluded if **Population:** urinary incontinence was caused by neurogenic disorders, gynaecology oncology or prostate cancer or pelvic organ disease or if participants were diagnosed with psychiatric disorders, mental illness, severe cognitive impairment, or end of life diagnosis. **Interventions:** were invasive conservative treatments, acupuncture, surgical or pharmacological interventions.

### Screening and study selection

Results of the five database searches were uploaded to Endnote software and duplicates were removed. Search results were then exported to Rayyan QRCI, where further de-duplication was performed. Articles were screened by title and abstract in Rayyan against the eligibility criteria by two independent reviewers (MOC and MS) (S1 Table). The remaining studies were then full text screened by two independent reviewers (MOC and MS), with any disagreements resolved in consultation with a third reviewer (AC)

### Data extraction

Descriptive data on the characteristics of each study, population, intervention (including the components of the multicomponent interventions), comparator, and outcome were extracted using a customised Excel template independently by two reviewers (MOC and AW) (S2 Table) and are presented in Tables 1 and 2. This review primarily aimed to evaluate the effects of interventions, and as such, we did not assess implementation fidelity or its impact on trial outcomes in the included studies. However, when studies reported on implementation fidelity, that information was extracted and is presented in Table 3. Implementation Fidelity and Implementation Strategies. Notably, only one study specifically targeted implementation fidelity by employing a quality assurance programme developed by the research team, however, implementation fidelity was not formally evaluated in that study [42]. The data was transferred into JASP software; a minor deviation from the PROSPERO protocol due to the expertise within the team in the use of JASP, which is comprehensive and user friendly [27]. JASP is an open-source platform similar to Cochrane Revman [28] and determines similar outputs.

**Table 1:** Characteristics of studies included in review.

Author, Year	Design	Country	Setting	Sample randomised (Intervention: Control Group) & Gender	Lost to follow up (Intervention: Control Group)	Age		Definition/ Descriptor of Frailty	Definition/Descriptor/Diagnosis of Urinary Incontinence
						(Mean ± SD/Median (IQR)			
						Conservative Intervention	Control		
Aslan et al, 2008	RCT	Turkey	1 nursing home	IG: 33 and CG: 31 All Female	IG: 8 and CG: 6	78.88 ± 4.80	79.44 ± 5.32	NH resident for > 6 months. Most had chronic illnesses.	Urinary wetting > few times/ month. UI assessed with ICS 1-hour pad test.
Hodl et al, 2019	Cluster RCT	Austria	12 Austrian Nursing Homes in 2 provinces	IG: 216 and CG: 165 All Female	36 dropouts- not differentiated between IG/CG	86	82.3	Dementia/ cognitive impairment diagnosis, mild to moderate score on the Care Dependency Scale.	UI described via questionnaire including prevalence, frequency, documented diagnosis, catheter, start of UI after admission
Hu et al, 1989	RCT	USA	7 Nursing Homes	IG: 72 and CG: 71 All Female	IG: 7 and CG: 3	85.6 ± 6.9	85.3 ± 7.7	NH residents with significant functional impairment (Katz ADL Score) and cognitive impairment (MMSE)	Inclusion criteria included daytime urinary incontinence and impairments in urodynamic studies in both groups at baseline
Kim et al, 2011	RCT	Japan	Community dwelling	IG: 31 and CG: 30 All Female	IG: 1 and CG: 1	79.0 ± 3.0	78.1 ± 4.4	Multiple symptoms of geriatric syndromes (MSGS) i.e., 2 of 3: urinary incontinence, functional decline, and fear of falling.	Frequency of UI ranging from 1 to 5 on a frequency scale (if scored from 2–5 on the scale, that is, from once or more/month to everyday) were defined as having UI.
Lai et al, 2017	RCT	Hong Kong	5 Nursing Homes (care and attention homes)	IG: 26 and CG: 26 Male & Female	IG: 0 and CG: 4	84.1 ± 10.4	86.7 ± 7.9	NH resident > 6 months, multimorbidity (total number of diseases), polypharmacy (> 5 medications), cognitive impairment (C-MMSE), functional impairment (Modified Barthel Index)	Stable rate of UI over the last 6 months, with UI defined as the presence of at least 2 episodes of urinary leakage per week in the previous 2 weeks
Lee et al, 2017	RCT	Korea	Community- dwelling	IG: 52 and CG: 46 All Female	IG: 10 and CG: 6	74.5 ± 4.1	75.6 ± 4.0	MCI/ Alzheimer’s Disease diagnosed by Neurologist/ Neuropsychologist, MMSE and Barthel’s ADL Index.	UI evaluated with a frequency volume chart (FVC) including a urinary sensation scale for urgency and the ICIQ-SF.
Schnelle et al, 2002	RCT	USA	4 Nursing Homes	IG: 94 and CG: 96 Male & Female	IG: 20 and CG: 22	87 ± 8	88 ± 7	Cognitive impairment (MMSE), high sickness severity; Cumulative Illness Rating Scale for Geriatrics (CIRS-G), multimorbidity, polypharmacy	Residents were identified as incontinent by Nursing Home staff
Schnelle et al, 2010	RCT	USA	6 Nursing Homes	IG: 65 and CG: 60 Male & Female	IG: 7 and CG: 6	85.8 ± 9.4	86.1 ± 10.5	NH Resident, cognitive impairment (MMSE), 95% needed a proxy to consent.	Residents were eligible if had UI and faecal incontinence
Suzuki et al, 2019	RCT	Japan	13 Nursing Homes	IG: 62 and CG: 57 Male & Female	IG: 12 and CG:27	84 (80-90)	86 (83 -89)	Care needs ≥ level 3, moderate severity of co-morbid disease (Charlson Co-morbidity Index), severe physical dependency (Barthel Index), cognitive impairment (MMSE), depression (GDS).	UI requiring use of pads by NH residents and residents having less than 300ml on their post-void residual volume test were the inclusion criteria.
Tak et al, 2012	RCT- multicentre	Netherlands	20 Homes for the Elderly	IG: 102 and CG: 90 All Female	IG: 17 and CG: 20	84.6 ± 6.5	84.7 ± 5.7	MCI (Cognitive Screening Test), mild physical disability (Barthel Index) and co-morbidities	Females with and without UI were included to avoid the stigma of UI when participating. Patients had to be able to toilet independently
van Houten et al, 2007	RCT	Netherlands	Nursing Homes, Homes for Elderly and Day Centres for elderly -nondemented	IG: 29 and CG: 28 All Female	IG: 3 and CG: 1	80.9 ± 7.35	84.5 ± 6.36	MCI (MMSE), mild to moderate physical impairment (Barthel Index), polypharmacy, all had mobility disorders	UI episodes of ≥ twice per week for at least 3 months. Type of incontinence classified by an algorithm based on a questionnaire concerning urge and stress test
Vinsnes et al, 2012	RCT	Norway	4 Nursing Homes, Norway	IG: 48 and CG: 50 Male & Female	IG: 13 and CG: 17	85.7 ± 8.2 overall – no differentiation between grps.		Resident in NH for > 3 months, needed assistance in at least 1 ADL, cognitive impairment on MMSE	Screened for UI with overall prevalence of UI of 89.7%.

ADL = Activities of Daily Living, CG = Control Group, GDS = Geriatric Depression Scale, grps. = groups, ICIQ-SF = International Consultation on Incontinence Questionnaire-Short Form, IG = Intervention Group, MCI = Mild Cognitive Impairment, MMSE = Mini Mental State Examination, NH = Nursing Home, RCT = Randomised Control Trial, UI = Urinary Incontinence

**Table 2:** Intervention components.

	Frequency		Delivered by	Format		Voiding Programme		Multicomponent Interventions															Staff Based Education	Comparator Intervention
Authors	Days per week	No. of weeks		Group	Individual	Prompted Voiding	Ultrasound Assisted PV	PFMT	Bladder Training	Education	Urge Suppression Tech.	Food Intake	Fluid Intake	Walking	Balance Training	Upper Body Strength.	Lower Body Strength.	Exercises in Sitting	ADL/Functional Training	Toileting Skills	Wheelchair Propulsion	Relaxation & Breathing		
Aslan et al, 2008	3 → 4	6 → 8	UG Nurse		√			√	√	√	√													Usual care
Hodl et al, 2019	NS^*^	13	^*^NH staff		√																		^†^ 29 guidelines	Usual care
Hu et al, 1989	7	13	NRAs		√	√																		Usual care
Kim et al, 2011	2	13	TMIG staff	√				√						√	√	√	√	√						Education
Lai et al, 2017	7	26	^*^ NH staff		√	√																		Usual care
Lee et al, 2017	Fort.	12	Expert PT		√			√	√	√	√													Education
Schnelle et al, 2002	5	32	Res. staff		√	√							√	√	√	√	√				√			Usual care
Schnelle et al, 2010	5	12	Res. Staff		√	√						√	√	√			√				√			Usual care
Suzuki et al, 2018	^*^NS	8	^*^NH staff		√		√																	CPV
Tak et al, 2012	1	22	Trained PT	√				√	√	√									√	√		√		Usual care
van Houten et al, 2007	3	1 → 8	PT/OT		√									√						√				Usual care
Vinsnes et al, 2012	5	13	PT & OT	√^*^	√									√	√	√	√		√					Usual care

*NS  = Not Specified Fort. = Fortnightly (2-week intervals) UG Nurse = Urogynaecological Nurse NRAs = Nursing Research TMIG = Tokyo Metropolitan Institute of Gerontology

*NH staff = trained Nursing Home staff PT = Physiotherapist Res. staff = Research staff OT = Occupational Therapist √^ *^ = FIT offered to individuals and groups PFMT = Pelvic Floor Muscle Training ADL = Activities of Daily Living Educ. Class = Education class CPV = Conventional Prompted Voiding Urge Suppresion Tech. = Urge Suppression Techniques Upper Body Strength. = Upper Body Strengthing Lower Body Strength. = Lower Body Strengthening

† 29 guidelines = PFMT, BT, Bladder diaries, lifestyle interventions (caffeine and fluid modifications, weight loss), MDT interventions, use of pads, urinals, toileting aids

**Table 3:** Implementation fidelity and implementation strategies.

Authors	Implementation Fidelity	Implementation Strategies					
	Yes/ No	Formal Implementation Blueprint ^*^	Clinical Practice Guidelines ^†^	Educational Meetings *†	Educational Materials *†	Continuous Quality Improvement ^†^	Identify and prepare champions ^*^
Aslan et al, 2008	No	Yes	No	No	Yes	No	No
Hodl et al, 2019	No	Yes	Yes	Yes	Yes	Yes	Yes
Hu et al, 1989	No	Yes	No	No	No	No	No
Kim et al, 2011	No	Yes	No	Yes	Yes	No	No
Lai et al, 2017	Yes °	Yes	No	Yes	No	Yes	No
Lee et al, 2017	No	Yes	No	Yes	No	No	No
Schnelle et al, 2002	No	Yes	No	No	No	No	No
Schnelle et al, 2010	No	Yes	No	No	No	Yes	No
Suzuki et al, 2018	No	Yes	No	Yes	No	No	No
Tak et al, 2012	No	Yes	No	Yes	Yes	No	No
Van Houten et al, 2007	No	Yes	No	No	No	No	No
Vinsnes et al, 2012	No	Yes	No	No	No	No	No

Yes ° = Fidelity addressed but not formally assessed

*= ERIC study

† = EPOC Taxonomy

### Quality assessment

Two independent reviewers (MOC and AW) assessed the methodological quality of included studies using the Cochrane Risk of Bias 2 tool [29]. Disagreements were resolved by consensus and the participation of a third arbitrator (AC) where necessary. Grading of Recommendations Assessments, Development and Evaluation (GRADE) analysis was applied to the outcomes to evaluate the quality of evidence and was completed by two independent reviewers (MOC and AW) (S3 Table) [28].

### Analysis

We provided a narrative synthesis of the findings from the included studies which did not meet the requirements to be combined in a meta-analysis [30]. The narrative synthesis was structured around the target population characteristics, type of intervention and intervention content and type of outcome.

Meta-analysis was run for each of the following outcomes with ≥ 3 independent samples: objective measures of UI and functional ability. Although two samples are sufficient for meta-analysis, unless these samples can be pooled meaningfully and have similar results, analysis of two samples results in low power [28]. Thus, for outcomes with ≥ 3 independent samples, post-treatment, standardised mean difference effects sizes (ESs) with confidence intervals (CIs) were calculated using Cohen’s *d* index of individual effects: *dk=(M1k–M2k)/S.D.pk*, where *d* is the effect size, *k* the individual sample, *M1k* intervention-group mean, *M2k* control-group mean and *S.D.pk* is the pooled standard deviation. Standardised ESs are necessary as despite studies assessing similar outcomes (e.g., UI objective and functional ability), different scales were used. Hedge’s *g* correction for small sample bias was then applied to ESs. These ESs were interpreted according to Cohen’s cut offs of 1.30 – very large, 0.80 – large, 0.50 – medium, and 0.20 – small ES [31].

Despite having a clearly defined research question, and clear inclusion and exclusion criteria all identified a priori to the review, substantial heterogeneity was still evident and so the data were pooled using a random effects model, while accounting for and further exploring heterogeneity. Samples were pooled and analysed using a random effects model on JASP software [27]. Random effects model assumes that different studies estimate different, yet related intervention effects, thus assigning more weight to larger samples [32]. As studies in this review varied in sample characteristics, heterogeneity will be substantial, justifying random effects. Clinical and methodological heterogeneity were identified in the interventions delivered, and the outcome measures used to assess effect, and so sub-group analysis or meta-regression to address statistical heterogeneity were not performed as there were insufficient studies for these methods to be executed. The *I*^*2*^ index was also examined to further quantify heterogeneity across the samples not otherwise due to chance. This was interpreted using recommended cut-offs: 25% small, 50% moderate, and 75% large heterogeneity [33]. To further address heterogeneity, two researchers rigorously checked the accuracy of the data extracted from the studies. A narrative synthesis was used to synthesise the findings where possible, acknowledging effect modifiers, to facilitate a meaningful interpretation of the pooled results from the studies which involved complex interventions in populations of older adults with frailty [28]. Finally, to address publication bias, Orwin’s Fail Safe *N* procedure was used to determine the number of unpublished studies with null effects that would reduce any significant findings to a trivial ES [34].

## Results

### Eligible studies

**Fig 1:**
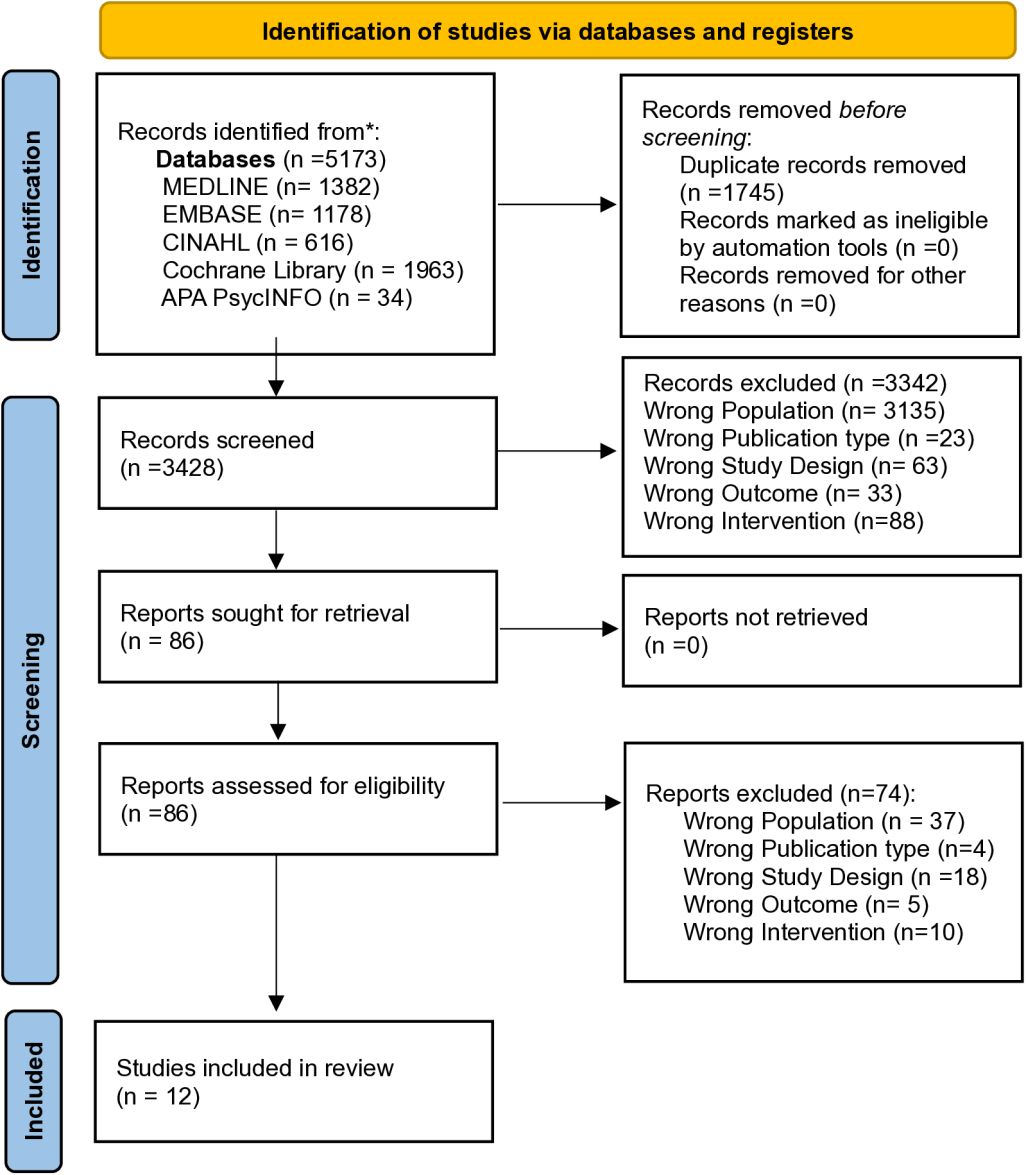
PRISMA 2020 Flow Diagram of study selection process.

The PRISMA Flow diagram Fig 1 displays the collection of studies included in the review. Initially, 4624 studies were identified, with a further 333 studies identified after re-running the search in May 2023, and a further 216 studies identified after re-running the search in April 2024, totalling 5173 studies identified, with twelve RCTs deemed eligible for inclusion.

### Characteristics of eligible studies

Table 1 presents the characteristics of the included studies. The studies were conducted across three continents: USA, Europe, and Asia. Ten of the twelve studies were conducted in residential care or nursing home settings and the remaining two studies were conducted in community settings [35,36]. Seven studies had all female participants [35–41] and five studies had mixed gender but majority female participants [42–46]. Participants mean age was greater than 80 years in nine studies and greater than 75 years in the other three studies [35–37]. Included studies were published from 1989 to 2019 (Table 1). Only two of the 12 studies reported specific conditions and concomitant diseases of the participants [35,36], including hypertension, stroke, and diabetes in one study [35], and mild cognitive impairment or mild Alzheimer’s disease in the other study [36]. None of the studies reported use of a standardised frailty assessment tool. Four of the twelve included studies did not have between group differences at baseline [35,37,43,46]. The other eight studies had between group differences at baseline, which will be discussed under the results of the relevant outcomes in the review.

### Quality of included studies

**Fig 2:**
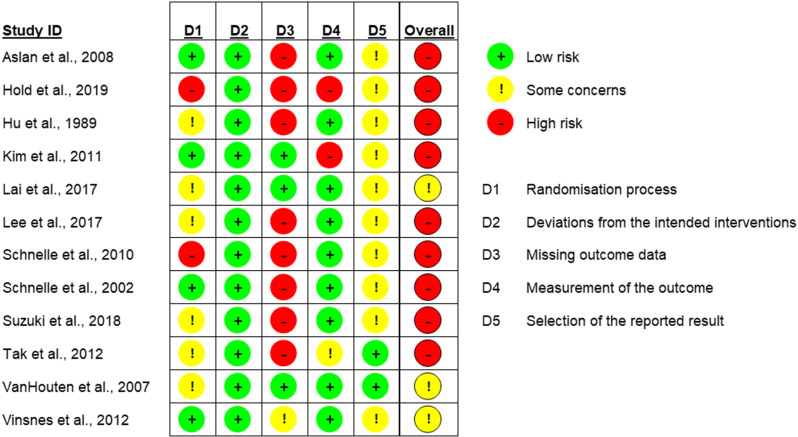
Risk of Bias Summary Cochrane RoB 2.0 Tool.

Nine studies were deemed to have a high risk of bias [35–40,43–45] and three were identified as having some concerns [41,42,46]. Results of quality appraisal are presented in Fig 2. As shown, bias was most commonly identified for Domain 3, due to missing outcome data.

### Urinary incontinence diagnosis

Two studies [36,37] used standardised measures to diagnose UI, the International Continence Society (ICS) 1 hour pad test and the International Consultation on Incontinence Questionnaire- Short Form (ICIQ-SF), respectively. Ten studies used a variety of non-standardised tools to diagnose UI, including informal screening of the participants to determine presence of UI [35,37,39–46], scales [35], questionnaire [38], urodynamic measures [39,45], frequency volume chart [36], and the need for pads to manage UI [45]. Five studies used more than one measure to diagnose UI [35–37,39,45] (Table 1).

### Interventions

Interventions were delivered on an individual basis to participants in all but three studies, where either group interventions [35,40] or the exercise component of the intervention was offered to individuals or groups [46].

The duration of the trials averaged 15 weeks (ranging from 6 to 32 weeks). The interventions were delivered by a range of professionals including internal trained nursing home staff [38,42,45], staff of a community gerontology centre [35], or external professionals including physiotherapists [36,40,41,46] and occupational therapists [41,46], a urogynaecology nurse [37], and research staff [39,43,44] (Table 2). The Cochrane Effective Practice and Organisation Care (EPOC) framework [47] and Expert Recommendations for Implementing Change (ERIC) [48] were used to categorise the implementation strategies employed in the studies included in the review (Table 3).

### Prompted voiding interventions

Prompted voiding interventions were provided as a single modality intervention in three studies [39,42,45]. Two studies demonstrated the effectiveness of (conventional) PV to usual care [39,42]. One study compared two active interventions, ultrasound-assisted PV and conventional PV, demonstrating that ultrasound-assisted PV was more effective than conventional PV [45]. Notably, significant differences in daytime urine loss were identified between the groups at baseline, which may have influenced the outcome [45].

### Interventions targeting nursing home staff

One study evaluated a comprehensive programme that included staff education and implementation of conservative management guidelines adapted from 29 recommendations from NICE, 2013 [49] and applicable to Austrian nursing home practice. The participants in the intervention group had tailored interventions that included strategies from a suite of options including lifestyle interventions (caffeine reduction, fluid modification, weight loss) behavioural and psychological interventions (bladder training, bladder diaries), physical interventions (PFMT), multidisciplinary team input and alternative conservative options (absorbent products, urinals, toileting aids) [38].

### Multicomponent interventions

Eight studies evaluated multicomponent interventions [35–37,40,41,43,44,46], which included a combination of different management strategies. Studies evaluating lifestyle interventions included one study encouraging increased fluid intake [43] and another study encouraged increased fluid and food intake [44]. Educational interventions included education of older adults as part of the treatment programme in three studies [36,37,40]. Behavioural and psychological interventions included prompted voiding in two studies [43,44], bladder training in three studies [36,37,40] and urge suppression techniques in two studies [36,37]. Physical interventions included pelvic floor muscle training in four studies [35–37,40] and exercise and physical activity interventions where most included walking [35,41,43,44,46], balance training [35,43,46], upper body strength training [35,43,46] and lower limb strength training [35,43,44,46]. Physical interventions also included three studies evaluating functional performance training exercises to increase independence in ADLs and toileting skills [40,41,46] (Table 2).

### Outcomes and outcome measures

A range of standardised and non-standardised assessment tools were used across the studies to diagnose UI and to measure changes in UI objectively and subjectively, patient and informal caregiver quality of life, functional ability, self-reported improvement in UI and adverse events. Eligible objective measures of urinary incontinence include wet checks, pad weight tests, frequency volume charts (FVCs), and bladder diaries [1]. UI is subjectively measured by patient reported outcomes such as questionnaires [1]. Functional ability consists of five domains of which ability to meet basic needs and mobility are relevant for this review [50]. The interventions recommended to improve mobility may be comprised of strengthening exercises, coordination, balance, aerobic fitness, and flexibility programmes [50]. Therefore, we included any validated activities of daily living (ADL) outcome measures (Barthel Index, Katz ADL Scale), mobility and physical capacity measures including walking measures, chair stands, balance, gait speed, muscle strength, hand grip strength and validated outcome measures (Physical Performance Test) in the Functional ability calculations. In the Functional Ability calculations, where ≥ one measure was reported by a study, an average of all the reported functional ability measures was taken and used to calculate the result.

All studies measured outcomes post intervention and three studies had an additional follow-up at either six months [35,37] or twenty-two weeks [39]. The primary outcomes of the trials are denoted by the symbol (¥) and the secondary outcomes by the symbol (φ) in Table 4. Outcomes and Outcome Measures. Four studies did not indicate whether the reported outcomes were primary or secondary [35,37,39,43]. Table 4 presents the quantitative findings across the studies showing the p value outcomes between groups in the studies, with one study showing within group p value outcomes [45].

**Table 4:** Outcomes and outcome measures.

Study	Outcome of Interest	Measurement Tool	Pre-Intervention		Post-Intervention		Outcome
Sample size (n)			IG (M ± SD)	CG (M ± SD)	IG (M ± SD)	CG (M ± SD)	p value
Aslan et al., 2008. Bladder training and Kegel Exercises for Women with Urinary Complaints Living in a Rest Home [37] **n = 64**	UI Objective Measures	Pad test,g	7.12 ± 12.07	8.20 ± 14.13	NR	NR	<0.001
	UI Subjective Measures	Daily urinary form	NR	NR	NR	NR	> 0.05
	Pelvic Floor Muscle Strength	Digital palpation	NR	NR	NR	NR	<0.001
	Quality of Life	King Health Questionnaire	NR	NR	NR	NR	< 0.05
Hodl et al., 2019. Effectiveness of conservative urinary incontinence management among female nursing home residents: A cluster RCT [38] **n = 381**	UI Subjective Measures ^¥^	Frequency of UI (questionnaire) ^¥^	53.2(%)	63.2(%)	NA (Odds Ratio)	NA (Odds Ratio)	0.02
	UI Objective Measures ^φ^	Received absorbent products^φ^	88.4(%)	90.9(%)	NA (Odds Ratio)	NA (Odds Ratio)	0.01
Hu et al., 1989. A Clinical Trial of a Behavioural Therapy to Reduce Urinary Incontinence in Nursing Homes [39] **n = 143**	UI Objective Measures	Wet episodes per day	2.22 ± 1.33	2.07 ± 1.07	1.65 ± NR	1.90 ± NR	<0.05
	Functional Ability	Katz Activities of Daily Living Scale	5.1 ± 1.5	5.5 ± 0.9	NR	NR	<0.05
Kim et al., 2011. The effects of multidimensional exercise on functional decline, urinary incontinence, and fear of falling in community-dwelling elderly women with multiple symptoms of geriatric syndrome: A randomized controlled and 6-month follow-up trial [35] **n = 61**	UI Subjective Measures	Frequency of UI (interview)	66.7(%)	51.7(%)	22.3(%)	44.8(%)	IG < 0.01; GG = 0.135
	Functional Ability (physical fitness tests)	One leg standing time (s)^†^	34 ± 24.2	33.4 ± 23.4	28.2 ± 20.4	28.8 ± 23.5	0.92
		Tandem walking (step)^†^	7.2 ± 4.7	7.8 ± 4.7	6.1 ± 4.5	5.2 ± 3.8	0.036
		Functional reach (cm)	31.7 ± 6.8	33.7 ± 4.7	33.5 ± 5.13	32.7 ± 5.3	0.046
		Grip strength (kg)^†^	17.2 ± 4	18 ± 4.6	20.9 ± 5.2	21.5 ± 5.1	0.874
		Adductor muscle strength (kg)^†^	17.2 ± 4	17.9 ± 5	18.9 ± 5.1	18.2 ± 4.01	0.045
		Usual walking speed (m/s)^†^	1.1 ± 0.3	1.2 ± 0.2	1.1 ± 0.2	1.1 ± 0.3	0.001
		Maximal walking speed (m/s)^†^	1.7 ± 0.4	1.7 ± 0.4	1.8 ± 0.5	1.6 ± 0.4	0.044
Lai et al., 2017. Using prompted voiding to manage urinary incontinence in nursing homes: Can it be sustained? [42] **n = 52**	UI Objective Measures ^¥^	Wet episodes per day*¥	3.7 ± 1.8	3.7 ± 1.2	3.1 ± 1.4	4.2 ± 1.3	0.001
		Incontinence rate per day %^¥^	61.6 ± 29 (%)	61.8 ± 19.3 (%)	52.5 ± 22.6(%)	70.4 ± 21.4(%)	0.001
		Total continent toileting/day^¥^	1.8 ± 2.5	1.7 ± 2.1	5.1 ± 2	1.6 ± 2.1	<0.001
Lee et al., 2017. Effects of pelvic floor muscle exercise on urinary incontinence in elderly women with cognitive impairment [36] **n = 98**	UI Subjective Measures and QOL measure ^φ^	ICIQ-SF ^φ^	11.8 ± 3.7	NR	9.2 ± 5.9	NR	<0.001
	UI Objective Measures ^¥^	UI episodes per day (Frequency Volume Chart)*¥	3.3 ± 1.1	3.4 ± 1.2	1.7 ± 1.	2.9 ± 1.1	<0.001
Schnelle et al., 2002. Translating Clinical Research into Practice: A Randomized Controlled Trial of Exercise and Incontinence Care with Nursing Home Residents [43] **n = 190**	UI Objective Measures	UI Frequency (% wet checks)^*^	37 ± 23 (%)	34 ± 21(%)	23 ± 21(%)	35 ± 21(%)	<0.01
		Appropriate toileting ratio (no. of times used toilet/no. of voids)	15 ± 23(%)	20 ± 28(%)	59 ± 34(%)	16 ± 25(%)	<0.01
	Functional Ability (behavioural observation and motion sensor data)	Average meters walked^†^	110 ± 99	140.8 ± 97.8	116.7 ± 92.6	105.6 ± 80.5	<0.01
		Average meters wheeled^†^	47.2 ± 34.6	41.7 ± 40.1	51.9 ± 38.4	28 ± 23.5	>0.05
		Maximum meters walked^†^	151.1 ± 117.4	199 ± 113.3	170.5 ± 107.7	169.2 ± 103.5	<0.01
		Maximum meters wheeled^†^	78 ± 44.4	79.4 ± 52.5	82.1 ± 52.2	57.7 ± 36.6	<0.05
		Average meters walk+wheeled^†^	92.9 ± 90.3	99.6 ± 92.6	98.3 ± 85.9	73.3 ± 73.8	<0.05
		Maximum meter walked + wheeled^†^	130 ± 106.9	150.4 ± 110.1	145.1 ± 102.9	123.9 ± 99.3	<0.01
		Average stands 30 seconds^†^	4.5 ± 2.5	5 ± 2.7	5.9 ± 2.6	4.6 ± 3.2	<0.01
		Maximum stands 30 seconds^†^	5.6 ± 2.6	6.4 ± 2.9	7.2 ± 2.8	6.3 ± 3.1	<0.05
		Stand level of assistance^†^	1.8 ± 1.1	1.7 ± 0.9	1.7 ± 0.7	2.2 ± 1.2	<0.01
		Arm raise^†^	8.6 ± 6.5	7.3 ± 4.5	12.6 ± 7.5	7.5 ± 4.6	<0.01
		Arm curl^†^	11.6 ± 7.7	10 ± 5.8	16.1 ± 8.7	11.3 ± 5.9	<0.01
Schnelle et al., 2010. A controlled trial of an intervention to improve urinary and faecal incontinence and constipation [44] **n = 125**	Lifestyle Intervention (Food/Fluid Intake)^φ^	Fluid intake (ounces/day)^φ^	0.66 ± 0.22	0.72 ± 0.18	13.5 ± 6.3	1.9 ± 4	
		Calories from snacks between meals^φ^	39.7 ± 76.6	32.9 ± 62.7	173 ± 152	67 ± 135	
	Functional Ability ^φ^	Sit-to-stands†φ	3.6 ± 3.1	2.1 ± 2	NR	NR	
		Walk or wheeled distance, meters†φ	58.7 ± 50.3	54.2 ± 55.8	59.9 ± 49.9	49.1 + 48.4	
	UI Objective Measures^¥^	Frequency of UI (% wet checks)^¥^	0.33 ± 0.2	0.33 ± 0.19	NR	NR	
		Appropriate toileting % (no. of voids in toilet/ total no. of voids)^¥^	0.19 ± 0.24	0.2 ± 0.27	NR	NR	
Suzuki et al., 2019. Ultrasound-assisted prompted voiding care for managing urinary incontinence in nursing homes: A randomized clinical trial [45] **n = 119**	UI Objective Measures (Frequency Volume Chart and pad weight test, g) ^¥^	Daytime voided volume, median (IQR), ml ^¥^	550(IQR 415–718)	550(IQR 340–670)	580(IQR 473–725)	540(IQR 375–700)	
		Daytime urine loss, median (IQR), g* ¥	300(IQR 195–470)	150(IQR 120–270)	250(IQR 100–438)	180(IQR 100–320)	
	Functional Ability ^φ^	Barthel Index, median IQR†φ	30(IQR 20–45)	45(IQR 35–55)	33(IQR 19–45)	45(IQR 30–51)	
	Quality of Life ^¥^	EQ-5D, median (IQR) ^¥^	0.533(IQR 0.204–0.605)	0.596(IQR 0.485–0.676)	0.533(IQR 0.200–0.613)	0.596(IQR 0.459–0.670)	
Tak et al., 2012. Does improved functional performance help to reduce urinary incontinence in institutionalized older women? A multicentre randomized clinical trial [40] **n = 192**	UI Objective Measures (Bladder Diaries)^¥^	UI episodes number/3 days*¥	8 ± 11	9.5 ± 11.5	9 ± 11.4	7.1 ± 9.6	
	Functional Ability^¥^	Physical Performance Test†¥	17.2 ± 4.87	15.8 ± 5.16	18.5 ± 4.14	14.7 ± 4.27	
	Quality of Life ^φ^	Health related quality of life (SF-12 mental)^φ^	47 ± 14.2	46 ± 12.8	52 ± 10	51.3 ± 8.5	
		Health related quality of life (SF-12 physical)^φ^	34.7 ± 12	34.1 ± 10.1	38.3 ± 11.6	35 ± 12.1	
		Sepcific quality of life (I-QOL)^φ^	68.9 ± 17.9	62.2 ± 17.7	65.7 ± 15.6	66.2 ± 15.6	
Van Houten et al., 2007. Urinary incontinence in disabled elderly women: A randomized clinical trial on the effect of training mobility and toileting skills to achieve independent toileting [41] **n = 57**	UI Objective Measures ^¥^	Pad test,g ^¥^	462(IQR 97–779)	448(IQR 88/736)	NR	NR	0.07
	Functional Ability ^φ^	Barthel Index ^φ^	13.4 ± 4.2	11.7 ± 3.6	NR	NR	>0.05
Vinsnes et al., 2012. Effect of physical training on urinary incontinence: a randomized parallel group trial in nursing homes [46] **n = 98**	UI Objective Measures ^¥^	Daily amount of leakage (24 PWT, grams)*¥	576 ± 465	424 ± 379	462 ± NA	653 ± NA	0.03

CG = Control group cm = centimetres IQR = Interquartile range IG = Intervention Group kg = kilograms M = Mean m/s = metres/second ml = millilitres NA = Not applicable NR = Not reported PA = Physical Activities 24 PWT = 24-hour pad weight test QoL = Quality of Life s = seconds SD = Standard Deviation

* = Used in Objective UI Meta-Analysis

† = Used in Functional Ability Meta- Analysis

¥ = Primary outcome of trial

φ = secondary outcome of trial

### Primary outcomes and outcome measures

#### 1. Objective measure of urinary incontinence.

**Fig 3:**
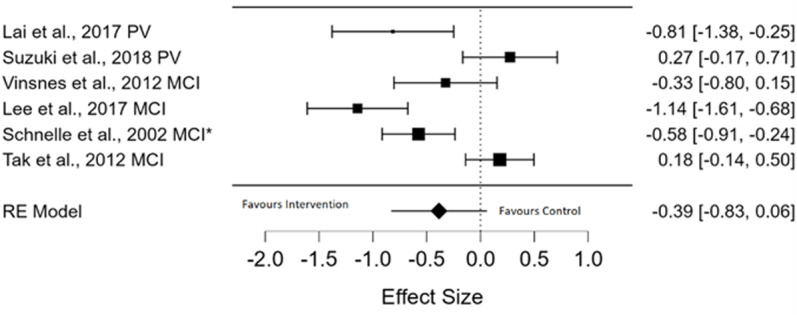
Forest Plot of Objective Measure of reduction in UI Meta-Analysis. PV, prompted voiding; MCI, multicomponent intervention; MCI*, multicomponent intervention + prompted voiding.

Eleven RCTs reported objective measures of UI, [36–46] (Table 4). Meta-analysis of six of these studies, pooled using a random effects model, showed no statistically significant improvement in objective measurement of UI (*g* = -0.39, *p* = 0.090; pooled effect size, with CI = - 0.39 [- 0.832, 0.060] and high levels of heterogeneity were observed (*I*^*2* ^= 85.26%)), (Fig 3) [36,40,42,43,45,46]. Effect sizes ranged from non-significant to very large. A GRADE assessment of the meta-analysed outcomes for objective measures of urinary incontinence showed that there is a very low certainty of evidence for these results (S3 Table).

Of the six studies included in the meta-analysis, two implemented prompted voiding (PV) as a single modality intervention, with mixed outcomes on the forest plot. PV resulted in a statistically significant reduction in objective UI, the primary outcome, measured using a weighed pad test, the incontinence rate per day, and the total continent toileting per day, between groups, at follow up, in favour of the IG [42]. The study reported that baseline between group difference in cognitive impairment did not influence the outcomes [42]. In contrast, in the second study, significant differences were identified between the groups at baseline and no significant effect was shown between ultrasound- assisted PV versus conventional PV, measured using a weighed pad test and frequency volume charts (FVC) [33]. However, the study identified a significantly beneficial within group effect [45].

The four other studies included in the meta-analysis implemented multicomponent interventions. One study implemented education (of patients), behavioural and psychological (BT, relaxation, and breathing) and physical interventions (PFMT, group functional exercises) but resulted in no reduction of objective UI between groups, measured with bladder diaries [40].The study identified a between group difference at baseline, with the CG participants experiencing a greater number of chronic illnesses than those in the IG [40]. In contrast, the other three studies that implemented multicomponent interventions reported no between group differences at baseline and statistically significant improvements in objective measures of UI, between groups, in favour of the IG, at follow up [36,43]. Of these three studies, one implemented lifestyle (fluid modification), behavioural and psychological (PV) and physical interventions (exercise) and used percentage wet checks of participants by staff and the appropriate toileting ratio [43]. The second assessed physical interventions (exercise, ADL and functional training) and used a weighed pad test [46]. The third assessed education (of patients), behavioural and psychological (BT, urge suppression techniques) and physical interventions (PFMT) using frequency volume charts (FVC) [36]. They reported within group significant reductions in UI in the IG and CG and between group differences in favour of the IG at follow up. The IG presented with a statistically significant higher duration of UI symptoms than the CG at baseline [36] (Table 4).

In the five studies not included in the meta-analysis due to insufficient data, 29 guidelines for conservative management of UI were implemented with significant reduction in use of absorbent products, between groups, in favour of the IG, at follow up [38]. There were between group differences, with a greater number of participants in the IG than CG, the IG participants were older, had a higher prevalence of UI and were more cognitively and physically frail than those in the CG at baseline [38]. Lifestyle (food and fluid modifications), behavioural and psychological (PV) and physical interventions (exercise) resulted in a statistically significantly improvement in the percentage of wet checks and the appropriate toileting percentage, between groups, in favour of the IG, at follow up [44]. There were between group differences at baseline, with statistically significant better cognitive function and physical ability in the IG than CG [44]. Education (of patients), behavioural and psychological (BT, urge suppression techniques, bladder diaries) and physical interventions (PFMT) resulted in a statistically significant improvement in weighed pad test, between groups, in favour of the IG, at follow up [37]. There were no between group differences at baseline [37]. Behavioural and psychological interventions (PV) resulted in a statistically significantly improvement in wet episodes per day within groups, in the IG and CG, and between groups in favour of the IG at follow up, with more cognitive impairment in the CG than IG at baseline [39]. Physical interventions (mobility and toileting skills programme) did not result in a statistically significant effect on weighed pad test, between groups at follow up [41]. There were between group differences at baseline, with the IG having better functional ability, more urge incontinence and younger participants than the CG [41] (Table 4).

#### 2. Subjective measure of urinary incontinence.

Four RCTs reported subjective measurement of UI as an outcome [35–38]. However, insufficient post-intervention data was reported in the studies and so a meta-analysis could not be run.

Multicomponent interventions included implementation of 29 guidelines for conservative management of UI in nursing homes resulting in a statistically significant reduction in subjective measures of UI via a non-standardised questionnaire, between groups, in favour of the IG, at follow up [38]. There were between group differences, with a greater number of participants in the IG than CG, the IG participants were older, had a higher prevalence of UI and were more cognitively and physically frail than those in the CG at baseline [38]. Physical interventions (PFMT, exercises) resulted in a statistically significant reduction in self- reported change during face-to-face interview, within group, in the IG only, at follow up and there were no between group differences at baseline [35]. Education (of patients), behavioural and psychological (BT, urge suppression techniques), and physical interventions (PFMT) resulted in a statistically significant reduction using a standardised UI questionnaire (ICIQ-SF), between groups, in favour of the IG, at follow up [36]. There was a between group baseline difference, with a statistically significant greater UI symptom duration in the IG [36]. Education (of patients), behavioural and psychological (BT, urge suppression techniques, bladder diaries) and physical interventions (PFMT) did not result in a significant improvement using a non-standardised daily urinary form, between groups and there were no between group differences at baseline [37] (Table 4).

#### 3. Patient Quality of Life (QoL).

Four RCTs reported QoL of participants [36,37,40,45] however insufficient data were available to assess using meta-analysis. Two studies showed statistically significant improvements in QoL; one study via a standardised UI questionnaire, ICIQ-SF [36] post implementation of a multicomponent intervention that included education (of patients), behavioural and psychological (BT and urge suppression techniques) and physical interventions (PFMT). Significant between group differences in QoL scores in favour of the IG were found at follow up and there was a between group baseline difference, with statistically significant greater UI symptom duration in the IG [36]. Another study used the UI- specific King’s Health Questionnaire (KHQ) post implementation of multicomponent interventions that included education (of patients), behavioural and psychological (BT, urge suppression techniques, bladder diaries) and physical interventions (PFMT) [37]. Significant within group and between group differences in QoL scores in favour of the IG were found at follow up and there were no between group baseline differences [37].

Conversely, no statistically significant improvement was found in QoL in the other two studies between groups at follow up. One implemented behavioural and psychological interventions (ultrasound-assisted PV), measured with the EuroQoL five dimensions questionnaire (EQ5D) and there were between group baseline differences, with significantly lower QoL scores in the IG than the CG [45]. The other study evaluated education (of patients), behavioural and psychological (BT, relaxation and breathing) and physical interventions (PFMT, functional training), measured with the Incontinence Quality of Life Instrument (I-QOL) and the 12 Item Short Form Survey (SF-12) [40]. This study had a between group baseline difference, with a greater number of chronic illnesses reported in the CG than the IG [40] (Table 4).

#### 4. Informal caregivers quality of life.

None of the included RCTs evaluated this outcome.

### Secondary outcomes and outcome measures

#### 1. Functional ability.

**Fig 4:**
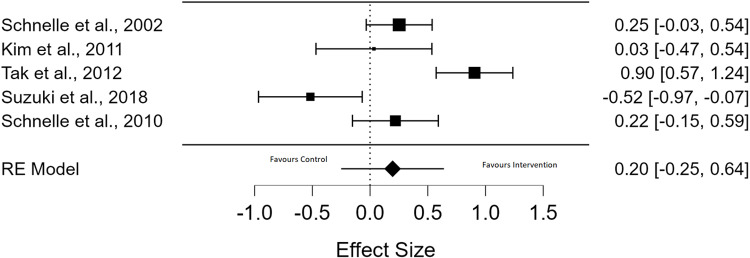
Forest Plot of Functional Ability Meta-Analysis.

Seven RCTs reported functional ability as an outcome [35,39–41,43–45]. Meta-analysis of five of these studies, pooled using a random effects model, showed no significant improvement in functional ability of participants in the intervention groups compared to controls (*g* = 0.20, *p* = 0.39, pooled effect size, with CI = 0.20 [- 0.251, 0.642] and high levels of heterogeneity were observed (*I*^*2* ^= 85.87%)) (Fig 4) [29,34,37–39]. Effect sizes ranged from non-significant to medium. A GRADE assessment of the meta-analysed outcomes for functional ability showed that there is a very low certainty of evidence for these results (S3 Table).

Of the five studies included in our meta-analysis, one implemented a behavioural and psychological intervention (PV) as a single modality, with more frequent toileting hypothesised to improve function, but resulted in no change in functional ability on the Barthel Index, either within or between groups at baseline [45]. The IG presented with significantly less functional ability than the CG at baseline [45]. In contrast, the four other studies that implemented multicomponent interventions were found to result in significant improvements in functional ability between groups, in favour of the IG, at follow up. One study implemented a physical intervention (multidimensional exercises), reported a statistically significant reduction in functional decline measured by physical fitness tests and did not have between group baseline differences [35]. A second study implemented lifestyle (fluid modification), behavioural and psychological (PV) and physical interventions (exercise), reported a statistically significant improvement in functional ability via behavioural observation and motion sensor data and no between group differences at baseline [43]. A third study implemented lifestyle (fluid and food modification), behavioural and psychological (PV) and physical interventions (exercise), reported a significant increase in physical activity but not endurance and there were between group baseline differences, with statistically significant better cognitive and physical function in the IG compared to the CG [44]. A fourth study implemented education (of patients), behavioural and psychological (BT, relaxation and breathing), and physical interventions (PFMT, functional training), reported a statistically significant increase in physical performance on the Physical Performance Test and between group baseline differences, with the CG participants experiencing a greater number of chronic illnesses than the IG [40] (Table 4).

In the two studies which were not eligible for meta-analysis, the first found that prompted voiding did not result in a significant between group difference at follow up using Katz’s Activities of Daily Living Scale and the CG presented with worse cognitive impairment at baseline compared to the IG [39]. In the second study, a training programme for mobility and toileting skills did not result in a significant difference between groups at follow up, using the Barthel index [41]. Baseline differences were reported between the groups, with the IG having better functional ability, more urge incontinence and younger participants than the CG [41] (Table 4).

#### 2. Self-reported improvement in UI.

One RCT reported this outcome [35] assessed via self- reported change during face-to-face interview. A statistically significant reduction was reported in the IG compared to the CG at follow up, and there were no between group baseline differences [35].

#### 3. Adverse events.

None of the included studies reported any adverse effects from the interventions.

### Other outcome measures reported in the included studies

One study evaluated pelvic floor muscle strength after a pelvic floor muscle intervention and found a statistically significant improvement between groups, in favour of the IG at follow up, with no between baseline group differences [37].

One study measured a change in food and fluid intake and reported a statistically significant improvement in both, between groups, in favour of the IG at follow up and there were between group baseline differences, with statistically significant better cognitive and physical function in the IG compared to the CG [44]. Another study encouraged fluid intake, but it was not measured as an outcome [43] (Table 4).

### Grading the evidence and summary of findings

For the primary outcome of improvement in objective measurement of urinary incontinence, the evidence was graded as very low. For the secondary outcome of improvement of functional ability with conservative non-pharmacological interventions the evidence was deemed as very low certainty (S3 Table).

## Discussion

Twelve primary RCTs investigating the effect of conservative non-pharmacological interventions on the management of urinary incontinence in older adults aged 65 years and over living with frailty with medium to high risk of bias were systematically reviewed and synthesised. This review had similar findings to a recent systematic review of systematic reviews of UI interventions in older adults by Kilpatrick et al, 2020, which encompassed 27 primary trials [19], even though the focus of our review was on older adults with frailty. Additionally, we included nine studies that were not included in the Kilpatrick review [19] due to our differing eligibility criteria and our focus on frail populations.

We found conservative interventions categorised as prompted voiding as a single modality intervention [42,45] and multicomponent interventions that included lifestyle interventions, educational interventions, behavioural and psychological interventions and physical interventions [36,40,43,46], did not result in a statistically significant reduction in objective measures of UI, when pooled in a meta-analysis, with very low certainty of evidence.

Multicomponent interventions implemented in the studies were found to have had mixed effects. Our findings were in line with the 7^th^ International Consultation on Incontinence (ICI) that reported increasing evidence of the effect of multicomponent interventions on UI in older adults with frailty, acknowledging that the evidence is insufficient as it is based on a pilot RCT [13], a RCT in nursing home residents aged > 60 years old [51] and two uncontrolled studies, which did not meet the criteria to be included in our review [1].

Prompted voiding (PV) resulted in improvements in objective measures of UI in two of our included studies as a single intervention [42,45]. These findings are in line with the findings of previous systematic reviews of conservative interventions for UI which included PV in older adults with frailty [23,52–55], a previous literature review [4], and the WHO evidence-based guidelines also recommend PV for older people with cognitive impairment [56]. The most recent ICI publication [1] recommended PV alone, or PV in combination with exercises that improve mobility and toileting, for UI in nursing home and home care residents, with ultrasound -assisted PV potentially more effective than conventional PV in nursing home clients [1]. Hence, the totality of evidence would suggest that conventional and ultrasound- assisted PV are beneficial as a treatment as a single modality intervention and as part of a multicomponent intervention to improve objective measures of UI.

Bladder training (BT) was included as part of a multicomponent intervention in three of our included studies [36,37,40] with two of these reporting a statistically significant reduction in objective measurement of UI [36,37]. BT is recommended for the treatment of urgency UI (UUI) [15,21] and for mixed UI (MUI) in the European Association of Urology (EAU) guidelines [15], as first line treatment for UI in women [16], with PFMT plus BT shown to be superior to BT alone for all types of UI in women [21]. A previous systematic literature review in community-dwelling older adults with frailty showed that multicomponent interventions including BT and Pelvic Floor Muscle Training (PFMT) were effective in reducing UI frequency based on the findings of three studies, one of which was a RCT [57]. Therefore, BT could be recommended in motivated patients with no significant cognitive or physical impairments [4], in line with ICI recommendations [1].

PFMT interventions are recommended as the first line of conservative treatment in women with UUI, SUI and all cause UI [21]. PFMT was included as part of multicomponent interventions in four of the included studies [35–37,40], with two studies with mixed effects for PFMT included in the objective reduction of UI meta-analysis [36,40] and the other two studies reporting a statistically significant reduction in subjective UI [35,36]. Therefore, the findings are mixed, and PFMT can be cautiously recommended in this population, if deemed appropriate as part of multicomponent treatment such as in older adults with frailty with good cognition [4] in line with guideline recommendations [1].

Five of the seven studies that evaluated the effectiveness of interventions on functional ability identified no significant improvement in the intervention group compared to controls when pooled in a meta-analysis [35,40,43–45]. Hence, the effectiveness of functional performance training on UI is equivocal.

However, as falls and UI are two of the five frailty syndromes [58] and research has shown that UI is associated with falls in older adults [59,60] and in frail older adults [61,62], a holistic approach to management is required. Research has identified a high prevalence of UI in older adults with functional impairments and fragility fractures post falls [63]. The benefits of exercise interventions for frail older adults are well established [64]. A recent network meta-analysis found that physical activity and resistance training in particular, along with multicomponent and nutrition interventions reduced frailty compared to controls [65]. There is conflicting evidence regarding the efficacy of multifactorial interventions in reducing falls in frail older adults [66,67], however, falls programmes should be modified and individually tailored for frail older adults to be effective [66]. Although this review did not demonstrate a statistically significant pooled effect of physical activity on objective measures of UI or functional ability, it is recommended that clinicians working with frail older adults deliver individually tailored interventions that preserve physical function and address physical frailty through multicomponent physical activity programmes [68] which may also reduce falls.

Three of the four studies which implemented multicomponent interventions reported a statistically significant reduction in subjective measures of UI [35,36,38]. However, one study noted that bladder diaries posed significant challenges for accurate completion by patients and nursing home staff, which led to missing outcome data [40].

UI is associated with poor QoL [69]. Of the four studies who reported QoL as an outcome, only two demonstrated a statistically significant improvement post intervention (multicomponent interventions) [36,37], measured by incontinence specific quality of life questionnaires; the ICIQ-SF and the King’s Health Questionnaire respectively. It is purported that an improvement was not found in the other two studies as they used generalised QoL tools, which may not have been sufficiently sensitive enough to measure a change [40,45].

The ICIQ-SF has been endorsed by the European Association of Urology (EAU) as a standardised measure to diagnose UI and a validated and reliable outcome measure to assess both symptoms and health related QoL (HRQoL) for UI in the general population [15]. However, as many factors contribute to UI in older adults with frailty [1], it is recommended that a validated UI-QoL measure specifically for older adults with frailty is developed [13] to include more relevant QoL domains like social interaction for nursing home residents or social participation for community dwelling frail older adults [1].

Overall, only two of the twelve included studies used standardised measures to diagnose UI, the ICS 1 hour pad test [37] and the ICIQ-SF questionnaire [36]. It is recommended that future studies use a standardised definition of UI to ensure accurate patient recruitment and comparison across research [1].

One of the primary outcomes of this review was informal caregivers’ quality of life but none of the included studies evaluated this outcome. It is well established that UI amongst older adults can lead to psychosocial, physical, and financial challenges for informal caregivers [70–72]. Thus, it is important that outcomes important to caregivers are measured in future trials.

Only two of the twelve studies implemented lifestyle interventions as part of a multicomponent intervention [43,44]. Malnutrition risk and frailty are interrelated and there is overlap in their definitions and diagnosis [73,74]. Thus, food and fluid-oriented lifestyle interventions could potentially target both frailty and malnutrition in older adults, addressing modifiable risk factors and co-morbidities to improve UI.

The effect of a multicomponent or a complex intervention will be determined by which combination of interventions are implemented, the mode of delivery, the healthcare professional delivering the interventions, the setting and the intensity of the programme [75]. Future research needs to establish the efficacy of tailored interventions that are designed to target individual needs to achieve maximum treatment success. In future research, multicomponent interventions need to target the causes of UI and any modifiable risk factors to ensure an individualised approach is taken to meet the needs of older frail adults with UI.

In this review, we reported the implementation strategies utilised in the included studies using relevant implementation strategies from both the EPOC [47] and ERIC [48] frameworks. Use of these taxonomies would enhance future implementation research and practice. Only one of our included studies targeted implementation fidelity by using a quality assurance programme, but implementation fidelity was not formally assed in the study [42]. There are many conceptual frameworks available to measure implementation fidelity [76], one being the Framework for Implementation Fidelity (FIF) [77], which could be used for future implementation research and practice. Future trials in this cohort could also utilise an effectiveness-implementation hybrid trial design [78], or embedded pragmatic trial (ePCT) design, in which onsite personnel, and not researchers deliver the interventions as per pre-planned process to measure outcomes [76]. The trial design ePCT, can be useful to understand variability in implementation fidelity, especially in multicomponent interventions delivered across multiple trial sites, such as across nursing home settings in a study [76].

International urology guidelines, European Association of Urology (EAU) guidelines [15] include some relevant recommendations for older adults but do not explicitly address frailty, and the NICE guidelines for management of UI in women do not mention older adults or older adults with frailty [15,16]. Thus, this systematic review adds to the existing body of literature as it synthesised the findings from RCTs with a focus on frailty, and this can help to inform clinical practice, future practice guidelines and future research trials of UI conservative management.

Several newer conservative non-pharmacological interventions were not represented in the trials included in our review including transcutaneous tibial nerve stimulation (TTNS). Two RCTs that evaluated TTNS [79] and TTNS and transcutaneous parasacral stimulation [80] identified mixed results. However, neither study was included in our review as participants were aged 60 years and over in both studies. These results may be in part explained by the differences between participants included in each trial.

Outcome heterogeneity underscores the need for development of a core outcome set for urinary incontinence in older adults with frailty. Core outcome sets (COS) are in development for studies with adults with stress urinary incontinence [81] and idiopathic OAB in women [82]. A COS for older adults with frailty and urinary incontinence is also needed given the unique outcome considerations compared to younger populations such as falls and functional ability. This recommendation aligns with the findings of the common data element and core outcome set for frailty which includes physical function and physical performance as core outcome domains [83].

The methodological quality of included RCTs was deemed poor overall, with nine studies scored as high risk of bias [35–40,43–45] and three as having some concerns regarding bias [41,42,46]. It is noteworthy that many of the included studies were well designed and implemented, however challenges with recruitment, assessments, outcome measures and high attrition due to intervening illness and death during trials influenced missing outcome data. This led to poor scores in Domain 3 of the RoB 2.0 Tool which resulted in overall judgements of high RoB in nine studies and moderate RoB in three included studies. These methodological flaws resulted in a low and very low certainty of evidence when assessing the results of our meta-analyses using GRADE. The factors that lowered the certainty of evidence included high risk of bias in nine of the twelve studies, inconsistencies in results reflected in the high heterogeneity scores in the meta-analyses, indirectness of evidence and imprecision due to relatively few patients and events, with resultant wide 95% CIs around the effect (S1 Table).

Reporting guidelines are now more robust and widely implemented and therefore it is easier to score well in the domains of the RoB 2.0 Tool in recent studies. Considering this, we recommend that future trials follow comprehensive standardised reporting guidelines. The RoB 2.0 Tool may not be as suitable for the older trials included in this review, but that does not mean that the studies nor their results are not meaningful and hold value in informing clinical practice. Pragmatic trials may be a more realistic study design to inform practice in a population with fluctuating levels of health status such as older adults with frailty [84]. Future evaluation of conservative interventions such as PFMT, BT or exercise interventions require selection of a dose, such as duration and intensity of training and may need to be pragmatic to carefully refine the intervention and assess its potential value in clinical practice.

### Strengths and limitations

Strengths were that a robust methodology was employed including the development of an a priori protocol, a comprehensive search strategy of databases and reference lists, the involvement of multiple researchers with their methodological and content expertise at each stage, and the use of Cochrane RoB 2.0 Tool and GRADE to determine the quality and strength of the evidence. A clear definition of frailty was outlined to inform inclusion/exclusion criteria and screening decisions in future trials to address the gaps identified.

There were also several limitations to this systematic review. Firstly, the significant heterogeneity in treatment strategies and outcome measures, and overall poor methodological quality of the included RCTs results in difficulties drawing firm conclusions from the current evidence base. Most studies included nursing home residents but there was significant heterogeneity within this population with regards to the criteria for admission to each facility. However, this is reflective of the population being studied. As observed across included studies, heterogeneity was high, and for some included studies, baseline group differences were noted, which may have impacted study outcomes. However, due to the limited number of studies eligible for the meta-analyses, sub-group analysis or meta-regression were not possible to explore further causes of heterogeneity. Future research should seek to explore if reported outcomes were moderated by various clinical and demographic characteristics.

The Cochrane Incontinence Group has since been archived, but we chose to use their categorisations as they were similar to the recent ICI guideline categorisation, and the interventions evaluated in our included RCTs aligned better with this system compared to the recent categorisations in the Cochrane overview of Cochrane systematic reviews for conservative management of UI in women aged ≥ 18 years old [21].

Only two of twelve studies included community dwelling frail older adults [35,36]. Worldwide, most frail older adults are community dwelling and so the findings from this systematic review may not be fully generalisable to these populations, who are likely to be less physically and cognitively frail than their counterparts who reside in long-term care. The search was limited to studies published in the English language, but no eligible trials were identified in other languages during our search. The review included limited evidence in men as studies did not include men at all or in those studies who did, men were the minority population.

There were limitations in that the number of eligible studies that we included was small, as studies did not quite meet the inclusion criteria. In one study, [85], 53% of participants were frail but our criteria were that 80% of a mixed population had to be frail as recommended by the Cochrane Handbook for Systematic Reviews [28]. We limited the age of included participants to 65 years and over as this matches the age range of our clinical population and the ICI definition of frail older persons [1]. However, it risks excluding potentially useful interventions in older adults with frailty who were less than 65 years old [86,87]. RCTs in community dwelling older adults with frailty including PFMT in community dwelling homebound frail older adults [88], behavioural therapy in homebound cognitively intact older adults [89] and a habit training programme in community-dwelling, caregiver-dependent older adults [90] were not included in this review as participants were < 65 years old. Lack of a clear definition of frailty in participants aged 65 years and over, also excluded studies implemented in community dwelling older adults, such as a multicomponent physiotherapy intervention [91] and PFMT in older women [92].

International standardised tools for assessing frailty, such as Fried’s Phenotypic Model and the Mitnitski and Rockwood cumulative deficits model, do not report specific health conditions. In our review only two studies reported participants health conditions or concomitant diseases [35,36]. Therefore, it is important in future studies on UI management in this population to use validated frailty tools to assess for frailty and include a detailed account of participants’ underlying conditions and comorbidities alongside their frailty grades, to aid interpretation of findings.

## Conclusion

The systematic review aimed to establish the effect of conservative non-pharmacological interventions on UI in older adults aged 65 years and over living with frailty. Twelve studies were included evaluating prompted voiding and multicomponent interventions including interventions targeting education of nursing home staff. Conservative non-pharmacological interventions had beneficial but not statistically significant effects on objective UI and functional ability. Meta- analysis of conservative interventions did not result in a statistically significant reduction in objective measures of UI when pooled, with very low certainty of evidence. Meta-analysis of five of seven studies reporting functional ability outcomes showed no significant improvement in the intervention groups compared to controls, with very low certainty of evidence. Only two studies reported a statistically significant improvement in patient QoL and none of the studies reported informal caregivers’ quality of life or adverse events. Further rigorous research is needed to establish optimal components of effective interventions, further effective interventions with a concomitant process for ensuring implementation fidelity and a core outcome set for studies with older adults living with frailty and UI. This is needed to ensure consistent evaluation of important outcomes such as patient and caregiver QoL.

## Supporting information

S1 File Search strategy. (DOCX)

S1 Appendix PROSPERO Protocol. (DOCX)

S1 Checklist PRISMA 2020 checklist. (DOCX)

S2 Checklist PRISMA 2020 for Abstracts checklist. (DOCX)

S1 Table Identified Studies. (DOCX)

S2 Table Data Extraction. (XLSX)

S3 Table GRADE Assessment of Meta-analysed Outcomes. (DOCX)

## Abbreviations

RCT: Randomised Controlled Trial
PRISMA: Preferred Reporting Items for Systematic Reviews and Meta-Analysis
GRADE: Grading of Recommendations Assessments, Development and Evaluation.
