# Supplementary material for: The effect of conservative non-pharmacological interventions on the management of urinary incontinence in older adults living with frailty: Systematic review and meta-analysis
Source: PLoS One. 2025 May 14;20(5):e0322742. doi: 10.1371/journal.pone.0322742 (PMC12077729; doi:10.1371/journal.pone.0322742)
Supplement: S1 File — (DOCX) [file pone.0322742.s001.docx]

**S1 File: Search Strategy**

**MEDLINE / APA PsycINFO/ CINAHL (EBSCO)**

| S49 | S44 *and* S45 *and* S46 | **Limiters** - English Language  **Expanders** - Apply equivalent subjects  **Search modes** - Boolean/Phrase |  |
| --- | --- | --- | --- |
|  | S48 | S44 AND S45 AND S46 |  |
|  | S47 | S44 AND S45 AND S46 |  |
|  | S46 | S41 OR S42 OR S43 |  |
|  | S45 | S27 OR S28 OR S29 OR S30 OR S31 OR S32 OR S33 OR S34 OR S35 OR S36 OR S37 OR S38 OR S39 OR S40 |  |
|  | S44 | S1 OR S2 OR S3 OR S4 OR S5 OR S6 OR S7 OR S8 OR S9 OR S10 OR S11 OR S12 OR S13 OR S14 OR S15 OR S16 OR S17 OR S18 OR S19 OR S20 OR S21 OR S22 OR S23 OR S24 OR S25 OR S26 |  |
|  | S43 | TI randomised controlled trial OR AB randomised controlled trial |  |
|  | S42 | TI (randomized control trial or RCT or randomised control trial or randomized controlled trial) OR AB (randomized control trial or RCT or randomised control trial or randomized controlled trial) |  |
|  | S41 | (MH "Randomized Controlled Trials as Topic+") OR "randomized controlled trial" |  |
|  | S39 | TI ((frail* N3 (#ld pe* or #ld adult* or syndrome))) OR AB ((frail N3 (#ld pe* or #ld adult* or syndrome))) |  |
|  | S38 | TI geriatric* OR AB geriatric* |  |
|  | S37 | TI senior* OR AB senior* |  |
|  | S36 | TI frail* OR AB frail* |  |
|  | S35 | TI (community dwelling older adults or elderly or seniors or geriatrics) OR AB ( community dwelling older adults or elderly or seniors or geriatrics ) |  |
|  | S34 | TI (care home or residential care or nursing home or residential home or long term care) OR AB (care home or residential care or nursing home or residential home or long term care) |  |
|  | S33 | TI vulnerable elderly OR AB vulnerable elderly |  |
|  | S32 | TI (frailty or frail elderly or vulnerable elderly or vulnerability or functionally impaired elderly) OR AB (frailty or frail elderly or vulnerable elderly or vulnerability or functionally impaired elderly) |  |
|  | S31 | TI frail old people OR AB frail old people |  |
|  | S30 | TI frail older adults OR AB frail older adults |  |
|  | S29 | (MH "Frail Elderly") OR "frail elderly" |  |
|  | S28 | (MH "Nursing Homes+") OR "nursing home" |  |
|  | S27 | (MH "Aged+") OR "aged" OR (MH "Aged, 80 and over") OR (MH "Homes for the Aged") |  |
|  | S26 | TI (pelvi* N5 rehabilit*) OR AB (pelvi* N5 rehabilit*) |  |
|  | S25 | TI ((void* N5 (prompt* or diar*))) OR AB ((void* N5 (prompt* or diar*))) |  |
|  | S24 | TI ((bladder N5 (train* or retrain*))) OR AB ((bladder N5 (train* or retrain*))) |  |
|  | S23 | TI (((bladder or detrusor) N5 (instab* or overactiv* or irritab*))) OR AB (((bladder or detrusor) N5 (instab* or overactiv* or irritab*))) |  |
|  | S22 | TI ((urin* N2 (leak* or urge* or frequen*))) OR AB ((urin* N2 (leak* or urge* or frequen*))) |  |
|  | S21 | TI (overactive bladder or overactive bladder syndrome or urgency or oab) OR AB (overactive bladder or overactive bladder syndrome or urgency or oab) |  |
|  | S20 | TI toilet* OR AB toilet* |  |
|  | S19 | TI detrusor instab* OR AB detrusor instab* |  |
|  | S18 | TI irritab* bladder OR AB irritab* bladder |  |
|  | S17 | TI urin* leak* OR AB urin* leak* |  |
|  | S16 | TI continen* OR AB continen* |  |
|  | S15 | TI incontinen* OR AB incontinen* |  |
|  | S14 | TI bladder control OR AB bladder control |  |
|  | S13 | TI sphincter incompetence OR AB sphincter incompetence |  |
|  | S12 | TI voiding dysfunction OR AB voiding dysfunction |  |
|  | S11 | TI urine leakage OR AB urine leakage |  |
|  | S10 | TI overflow incontinence OR AB overflow incontinence |  |
|  | S9 | TI mixed urinary incontinence OR AB mixed urinary incontinence |  |
|  | S8 | TI urinary continence OR AB urinary continence |  |
|  | S7 | (MH "Urinary Bladder, Overactive") OR "overactive bladder" |  |
|  | S6 | (MH "Pelvic Floor") OR "pelvic floor" |  |
|  | S5 | (MH "Toilet Training") OR "toilet training" |  |
|  | S4 | (MH "Urinary Bladder") OR "urinary bladder" |  |
|  | S3 | (MH "Urinary Incontinence, Urge") OR "urge incontinence" |  |
|  | S2 | (MH "Urinary Incontinence, Stress") OR "stress incontinence" |  |
|  | S1 | (MH "Urinary Incontinence+") OR "urinary incontinence" OR (MH "Urinary Incontinence, Urge") OR (MH "Urinary Incontinence, Stress") OR (MH "Nocturnal Enuresis") OR (MH "Diurnal Enuresis") |  |

**OVID Search strategy (Embase)**

1     exp urine incontinence/

2     exp frail elderly/

3     exp frail elderly/ or exp frailty/

4     1 and 3

5     limit 4 to randomised controlled trial

6     incontinen*.mp.

7     continen*.mp.

8     urinary [incontinence.mp](http://incontinence.mp/).

9     urge [incontinence.mp](http://incontinence.mp/).

10   stress [incontinence.mp](http://incontinence.mp/).

11   mixed urinary [incontinence.mp](http://incontinence.mp/).

12    overflow [incontinence.mp](http://incontinence.mp/).

13  (bladder adj5 (overactiv* or irritab*)).mp.

14 (detrusor adj5 instab*).mp.

15  voiding [dysfunction.mp](http://dysfunction.mp/).

16 urin* leak*.mp.

17  sphincter [incompetence.mp](http://incompetence.mp/).

18 toilet$.mp.

19 (bladder adj5 (train* or retrain$)).mp.

20 (void$ adj5 (prompt$ or diar$)).mp.

21 (urin$ adj2 (leak$ or urge$ or frequen$)).mp.

22 (pelvic adj5 rehabilit$).mp.

23    toilet [training.mp](http://training.mp/). or exp toilet training/

24    pelvic [floor.mp](http://floor.mp/). or exp pelvis floor/

25    6 or 7 or 8 or 9 or 10 or 11 or 12 or 13 or 14 or 15 or 16 or 17 or 18 or 19 or 20 or 21 or 22 or 23 or 24

26     limit 25 to aged <65+ years>

27     limit 26 to (Human and English language)

28     limit 27 to randomized controlled trial

29     frail [elderly.mp](http://elderly.mp/).

30     frail [older.mp](http://older.mp/).

31     [aged.mp](http://aged.mp/).

32     frail*.mp.

33     nursing [home.mp](http://home.mp/).

34     vulnerable [elderly.mp](http://elderly.mp/).

35     geriatric*.mp.

36     senior*.mp.

37     29 or 30 or 31 or 32 or 33 or 34 or 35 or 36

38     limit 37 to aged <65+ years>

39     limit 38 to (Human and English language)

40     limit 39 to randomized controlled trial

41     25 and 37

42     limit 41 to aged <65+ years>

43     limit 42 to (Human and English language)

44     limit 43 to randomised controlled trial

45     28 and 40

46     [faecal.mp](http://faecal.mp/).

47     [fecal.mp](http://fecal.mp/).

48     46 or 47

49     45 not 48

50     [surgery.mp](http://surgery.mp/). or exp surgery/

51     49 not 50

52     [medication.mp](http://medication.mp/). or exp drug therapy/

53     51 not 52

**Cochrane Search Strategy**

#1 MeSH descriptor: [Urinary Incontinence] explode all trees

#2 MeSH descriptor: [Urinary Incontinence, Stress] explode all trees

#3 MeSH descriptor: [Urinary Incontinence, Urge] explode all trees

#4 MeSH descriptor: [Urinary Bladder, Overactive] explode all trees

#5 MeSH descriptor: [Urinary Bladder] explode all trees

#6 MeSH descriptor: [Toilet Training] explode all trees

#7 MeSH descriptor: [Pelvic Floor] explode all trees

#8 ("urinary incontinence"):ti,ab,kw (Word variations have been searched)

#9 (mixed urinary incontinence):ti,ab,kw (Word variations have been searched)

#10 ("overflow incontinence"):ti,ab,kw (Word variations have been searched)

#11 (urine leakage):ti,ab,kw (Word variations have been searched)

#12 ("voiding dysfunction"):ti,ab,kw (Word variations have been searched)

#13 (sphincter incompetence):ti,ab,kw (Word variations have been searched)

#14 (bladder control):ti,ab,kw (Word variations have been searched)

#15 ("overactive bladder"):ti,ab,kw (Word variations have been searched)

#16 (incontinen*):ti,ab,kw (Word variations have been searched)

#17 (continen*):ti,ab,kw (Word variations have been searched)

#18 (urin* leak*):ti,ab,kw (Word variations have been searched)

#19 (irritab* bladder):ti,ab,kw (Word variations have been searched)

#20 (detrusor instab*):ti,ab,kw (Word variations have been searched)

#21 (toilet*):ti,ab,kw (Word variations have been searched)

#22 ("overactive bladder syndrome"):ti,ab,kw (Word variations have been searched)

#23 ((urin* NEXT 2 (leak* or urge* or frequen*))):ti,ab,kw (Word variations have been searched)

#24 (((bladder or detrusor) NEXT 5 (instab* or overactiv* or irritab*))):ti,ab,kw (Word variations have been searched)

#25 ((bladder NEXT 5 (train* or retrain*))):ti,ab,kw (Word variations have been searched)

#26 ((void* NEXT 5 (prompt* or diar*))):ti,ab,kw (Word variations have been searched)

#27 ((pelvi* NEXT 5 rehabilit*)):ti,ab,kw (Word variations have been searched)

#28 MeSH descriptor: [Aged] explode all trees

#29 MeSH descriptor: [Nursing Homes] explode all trees

#30 MeSH descriptor: [Frail Elderly] explode all trees

#31 (frail older adults):ti,ab,kw (Word variations have been searched)

#32 (frail older people):ti,ab,kw (Word variations have been searched)

#33 ("frailty"):ti,ab,kw (Word variations have been searched)

#34 (vulnerable elderly):ti,ab,kw (Word variations have been searched)

#35 (care home):ti,ab,kw (Word variations have been searched)

#36 (residential care):ti,ab,kw (Word variations have been searched)

#37 (community dwelling older adults):ti,ab,kw (Word variations have been searched

#38 (frail*):ti,ab,kw (Word variations have been searched)

#39 (senior*):ti,ab,kw (Word variations have been searched)

#40 (geriatric*):ti,ab,kw (Word variations have been searched)

#41 ((frail* NEXT 3 (#ld pe* or #ld* adult* or syndrome))):ti,ab,kw (Word variations have been searched)

#42 ((vulnerab* NEXT 3 (pe* or #lder*))):ti,ab,kw (Word variations have been searched)

#43 MeSH descriptor: [Randomised Controlled Trial] explode all trees

#44 ("randomised controlled trial"):ti,ab,kw (Word variations have been searched)

#45 ("randomised control trial"):ti,ab,kw (Word variations have been searched)

#46 ("randomised controlled trial"):ti,ab,kw (Word variations have been searched)

#47 ("randomised control trial"):ti,ab,kw (Word variations have been searched)

#48 #1 or #2 or #3 or #4 or #5 or #6 or #7 or #8 or #9 or #10 or #11 or #12 or #13 or #14 or #15 or #16 or #17 or #18 or #19 or #20 or #21 or #22 or #23 or #24 or #25 or #26 or #27

#49 #28 or #29 or #30 or #31 or #32 or #33 or #34 or #35 or #36 or #37 or #38 or #39 or #40 or #41 or #42

#50 #43 or #44 or #45 or #46 or #47

#51 #48 AND #49 AND #50 1847
