# Supplementary material for: The effect of conservative non-pharmacological interventions on the management of urinary incontinence in older adults living with frailty: Systematic review and meta-analysis
Source: PLoS One. 2025 May 14;20(5):e0322742. doi: 10.1371/journal.pone.0322742 (PMC12077729; doi:10.1371/journal.pone.0322742)
Supplement: S1 Appendix — (DOCX) [file pone.0322742.s002.docx]

## S1 Appendix: PROSPERO Protocol

Details of the protocol for this systematic review were registered on PROSPERO and can be accessed at: <https://www.crd.york.ac.uk/prospero/>display_record.php?ID=CRD42022316287

**Updates to the PROSPERO protocol**

Overall, no significant changes were made to the PROSPERO protocol as published, only the use of JASP software in lieu of Cochrane RevMan for data analysis. PROSPERO requested an update on the status of the review in February 2024 and this was completed.
