# Supplementary figures and images for: The effect of conservative non-pharmacological interventions on the management of urinary incontinence in older adults living with frailty: Systematic review and meta-analysis
Source: PLoS One. 2025 May 14;20(5):e0322742. doi: 10.1371/journal.pone.0322742 (PMC12077729; doi:10.1371/journal.pone.0322742)

**S2 Checklist: PRISMA 2020 Abstract Checklist**
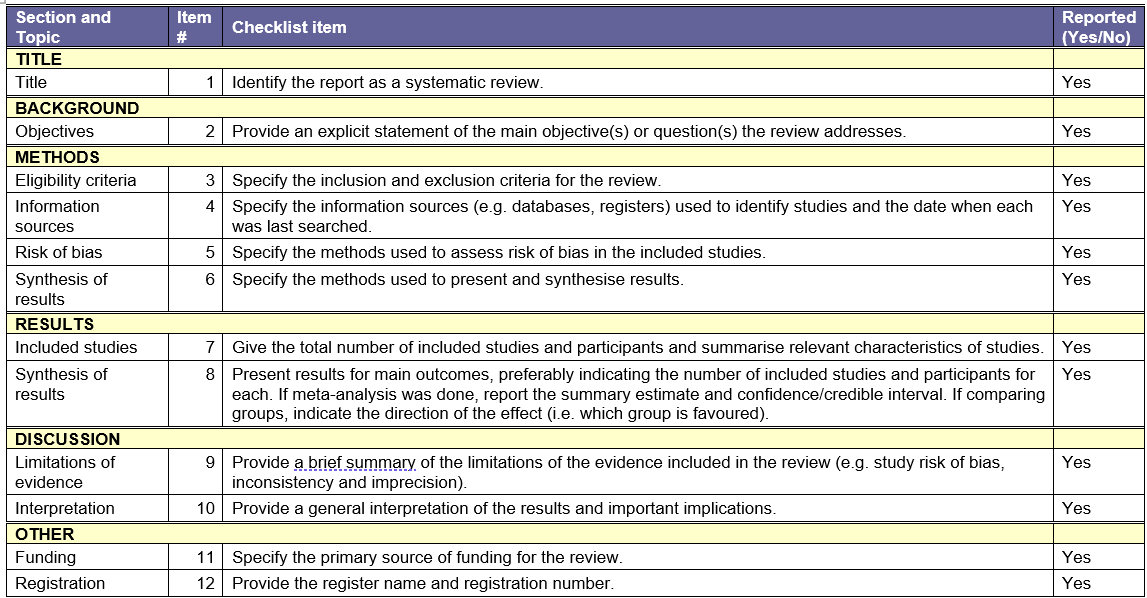

Supplement: S2 Checklist — (DOCX) [file pone.0322742.s004.docx]

**S3 Table: GRADE Assessment of Meta-Analysed Outcomes**

**
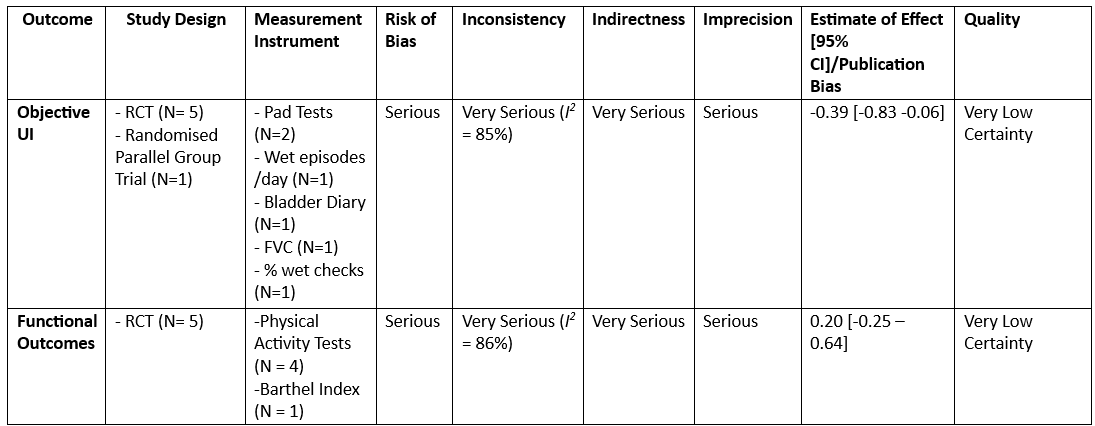
**

Supplement: S3 Table — (DOCX) [file pone.0322742.s007.docx]
